# Serological Evaluation of EgAgB16 kDa, a Recombinant Antigen from *Echinococcus granulosus* for Diagnosis of Human Hydatidosis

**Published:** 2010-09

**Authors:** J Abdi, B Kazemi, A Haniloo, M Mohebali, M Mahmoudi, S Rezaei, M Bandehpour, L Maghen, MB Rokni

**Affiliations:** 1 Department of Medical Parasitology & Mycology, School of Public Health, Tehran University of Medical Sciences, Tehran, Iran; 2 Department of Parasitology, Shahid Beheshti University, M.C., Tehran, Iran; 3 Cellular and Molecular Biology Research Center, Shahid Beheshti University, M.C., Tehran, Iran; 4 Department of Medical Parasitology & Mycology, School of Medicine, Zanjan University of Medical Sciences, Zanjan, Iran; 5 Department of Epidemiology and Biostatistics, Medical Parasitology & Mycology, School of Public Health, Tehran University of Medical Sciences, Tehran, Iran

**Keywords:** Hydatidosis, *Echinococcus granulosus*, ELISA, Recombinant, Antigen

## Abstract

**Background:**

Regarding that accurate diagnosis of human hydatidosis still needs more investigations, the present study was conducted to clone, express, and evaluate the gene encoding AgB subunits (EgAgB16 kDa) from *Echinococcus granulosus* (Iranian G1 strain) and its evaluation by ELISA test.

**Methods:**

DNA was extracted from protoscoleces and was utilized by PCR for strain identification. Total RNA was prepared with RNeasy protect mini kit from *E. granulosus* (Iranian G1 strain) protoscoleces collected from naturally infected sheep with hydatid cyst. Recombinant AgB16 kDa was produced using pETDuet as vector and evaluated by ELISA method. A panel of sera including hydatid cyst-infected individuals (n=72), healthy individual (n=48), toxoplasmosis (n=4), strongyloidosis (n=4), kala-azar (n=5) and tuberculosis (n=5) were examined using this recombinant antigen.

**Results:**

Recombinant protein was purified by affinity chromatography using His-Tag column. After purification, recombinant protein was confirmed by western blot analysis using His Tag monoclonal antibody or hydatid positive human serum. The sensitivity, specificity; positive and negative predictive values were calculated as 93.5%, 95.6%, 96% and 92.9%, in that order. The cut-off point was detected 0.3 for rAgB16.

**Conclusion:**

While the produced recombinant AgB16 kDa showed promising results in diagnosing human hydatidosis, but more investigations should be implemented to reach an accurate gold standard.

## Introduction

One of the most important and fatal helminthic diseases, caused by the larvae of the parasite, not the adult itself, is cystic echinococcosis or hydatid disease. It is a larval stage of *Echinococcus granulosus*, with canids as definite host and is a zoonotic disease, which not mention of human can infect a wide variety of animals including ruminants, carnivorous etc as intermediate host (1). The disease has been classified as food borne disease by WHO and is transmitted to humans by ingesting the parasites’ eggs through geophagia, contact with dog, eating vegetable and so on (2). It is a disease of very long prepatent period and needs a long period to be discovered by the physician. The estimated human burden of disease is reported as 285,407 DALYs and an annual loss of US $193,529,740 (3).

Regarding the widespreading of hydatidosis throughout the world, the diagnosis of the disease is still a matter of challenge. The reason is the biology of the disease and the lack of an authentic gold standard, being accepted by all researchers (1). Iran is a country of endemic situation for hydatidosis (2, 4) and regardless of utilizing ELISA test for its diagnosis in some medical labs, still encompasses the problem of diagnosing the disease in a 100% certainty (2). At present a mixture of imaging techniques besides of immunological tests are implemented to reach a conclusive diagnosis.

To assist fixing the issue of this dilemma, many studies have been conducted so far and a wide variety of immunological techniques have been evaluated so far, mostly based on ELISA test (5–9). The most challenging point for these researchers has been to establish and evaluate a new antigen of the parasite, which can improve the validity of the test as far as possible. In this regard, the present authors could clone, express, and evaluate an antigen B subunit entitled EgAgB12 kDa in GenBank, from *E. granulosus* (Iranian G1 isolates) in *E. coli* and evaluate it by ELISA (In press). Before that a part of the present team had produced recombinant EgAgB (24 kDa) and evaluated it by ELISA test (10).

Many researchers have reported findings on producing and evaluating different recombinant antigens form *E. granulosus* so far (11–13). Obviously, nobody can deny the important role of specific strains of each region in determining the value of the produced recombinant antigen, so we believe that each strain form different countries must be challenged in appraising new antigens.

Reaching a far more efficient antigen and clearing all issues of diagnosing the disease persuaded us in this study to clone, express, and evaluate the gene encoding AgB subunits (EgAgB16 kDa) from *E. granulosus* (Iranian G1 strain) in *Escherichia coli* (TΔM15alfa).We evaluated the antigen by ELISA test and compared the results with previous findings.

## Materials and Methods

### Clinical samples

A panel of sera including hydatid cyst-infected individuals (n=72), healthy individual (n=48), toxoplasmosis (n=4), strongyloidosis (n=4), kala-azar (n=5) and tuberculosis (n=5) from the Tehran School of Public Health serum blood bank were obtained. Hydatidosis had been confirmed by surgery as well as pathology and the rest of the infections were approved by different tests including ELISA, IFA, and authentic methods normally being utilized in routine diagnosis of different parasitoses in our reference center. Healthy controls were from those volunteers at the school. An informed consent was given from each patient and the integrity of the study was approved by the human Ethics Committee at the School of Public Health, Tehran University of Medical Sciences, Iran.

### Strain identification

DNA was extracted from protoscoleces and was utilized by PCR with the specific PCR primers as F: 5′-GCT TTT GTG TGG ATT ATG CG-3′and 5′-TCA AAC CAG ACA TAC ACC AA-3′. PCR was performed using 0.5 µl of 10 mM dNTP, 20 pmol of each primers and 1.25 U Taq DNA polymerase (CinnaGen, Tehran, Iran) in a total volume of 30 µl. The amplification reaction was carried out in a programmable thermal controller thermocycler (Eppendorf, Homburgs**,** Germany) as follows: 35 cycles at 98 °C 30 s, 57 °C 30 s and 72 °C 40 s followed by a final extension at 72 °C for 5 min. The 259 bp was produced. Primers specifically amplified portions of the mitochondrial 12SrRNA gene of the G1 strain of *E. granulosus* already recognized in Iran (14).

### RNA Extraction, cloning, and gene expression

Total RNA was prepared with RNeasy protect mini kit (Qiagene, Las Matas, Spain) from *E. granulosus* (Iranian G1 strain) protoscoleces. First strand cDNA was synthesized using reversetranscriptase (Roche Pharma, Spain). Briefly, cDNA was synthesized with total RNA at a final reaction volume of 20 µl containing 2 µl of 10× buffer RT, 0.5 mmol/l of each dNTP, 1 µmol/l of random primers (Promega, Duebendorf, Switzerland), 10 U RNase inhibitor and 4 U Omniscript Reverse Transcriptase. Following incubation for 2 h at 37 °C, Omniscript Reverse Transcriptase was inactivated by heating the mixture to 93 °C for 5 min.

Produced total AgB and HydI cDNA were utilized by PCR with the specific PCR primers EgAgB F (5-AAG CTT ATG CTT CTC GCT CTG GCT C-3) and EgAgB R (5-CTC GAG CTA TTT ACCTTC AGC AAC C-3) and hydI F:5′- GGA TCC ATG AGG ACT TAC ATC CTT C-3′ and HydI R:5′-AAG CTT TGA ATC ATC ATT C-3′ which were based on the nucleotide sequence from antigen B and HydI and were available at GenBank (accession number AgB**: M36774** and HydI:DQ835667). These primers contained HindIII and xhoI restriction sites for AgB and BamHI and HindIII restriction sites for HydI to facilitate subsequent cloning steps.

PCR was performed using 0.5 µL of 10 mM dNTP, 20 pmol of each primers and 1.25 U Taq DNA polymerase (CinnaGen, Tehran, Iran) in a volume of 30 µl for B and HydI genes. The amplification reactions was carried out in a programmable thermal controller thermocycler (Eppendorf, Homburgs, Germany) as follows: 30 cycles at 98 °C 30 s, 50 °C 30 s for AgB and 57 °C 30 s for HydI and 72 °C 30 s followed by a final extension at 72 °C for 5 min. The 231 bp and 276bp amplification products were gel purified (gel extraction kit, Fermentas, Vilnius, Lithuania). The purified fragments (AgB:231bp and HydI:276bp) sequenced at the automated sequence service were cloned into T-vector, PTZ57R (Invitrogen, Barcelona, Spain) using T4 DNA ligase separately. Reactions were transformed into *E. coli* competent cells, and distributed on LB agar plate containing 100 µg/ml ampicillin, X-gal and IPTG. White bacterial colonies were screened as containing recombinant plasmids. Selected colonies were mass cultured and plasmid was extracted using alkaline method. Recombinant plasmids were digested with restriction enzyme (HindIII and xhoI for AgB and BamHI and HindIII for HydI) (Fermentas, Vilnius, Lithuania), released fragments (AgB and HydI) were purified, and electrophoresed, and then at first the resulting products (AgB) was subcloned into PET-Duet expression vector with T4 DNA ligase (Roche Pharma, Pleasanton, US). After Subcloning of AgB into expression vector and its confirmation, HydI gen was subcloned into those expression vector (PET-Duet) that had a AgB gene. Therefore, AgB and HydI had been subcloned into one expression vector serially to production of recombinant protein.

### Protein purification

The recombinant protein was purified by affinity chromatography, based on its *N*-terminal His tag using Ni-NTA His-bind resin (15). Purity and integrity of recombinant protein were checked by 15% polyacrylamid gel stained with Coomassie brilliant blue G250. Antigen concentration was determined by biophotometer (Eppendorf, Hamburgers, Germany). Protein was stored at −80 °C until use.

### SDS-PAGE and Immuonoblotting

Bacterial cell lysate was electrophoresed on 15% SDS-polyacrylamide gels and blotted onto PVDF (Polyvinilidine difluoride) membrane (U-CyTech Elispot kits & Reagents from Aniara). Membrane was probed with hydatid positive human serum antibody and detected with a dilution of 1:100 pool of five human CHD sera and with antibody anti rAg diluted 1:500. Goat anti human immunoglobulin IgG conjugated to horseradish peroxidase, as well as diaminobenzidin/H2O2 was used to visualize the antigen antibody reaction.

### ELISA

ELISA was conducted as reported earlier (16). The diluted antigen in carbonate/bicarbonate sodium buffer as 2 µg/ml for rAgB16 kDa was coated on the plates and incubated overnight at 4 °C. After washing three times with PBS-Tween 20, and blocking the wells using bovine serum albumin 3%, and another one-stage washing, the 200-fold diluted serum samples were incubated over the coated antigen for 1 hour at 37 °C. After washing and addition of the conjugate (Sigma immunochemical St. Louis MO) at a dilution of 1/10000 of the anti-human IgG, and incubation for 1 hour at 37 °C, the plates were washed six times, the substrate Orthophenylendiamine (Sigma immunochemical St. Louis MO) was added, incubated for 10 min and stopped by addition of 0.5 M H_2_SO_4_. The OD of the reaction was measured at 492 nm with a 620 nm reference filter with a Tekan ELISA reader.

### Statistical analysis

The cut-off value was calculated as the mean plus 3.0 standard deviations of the OD value obtained for sera from healthy participants. The sensitivity, specificity, positive and negative predictive values were calculated with the method of Galen (17).

## Results

**Fig. 1:**
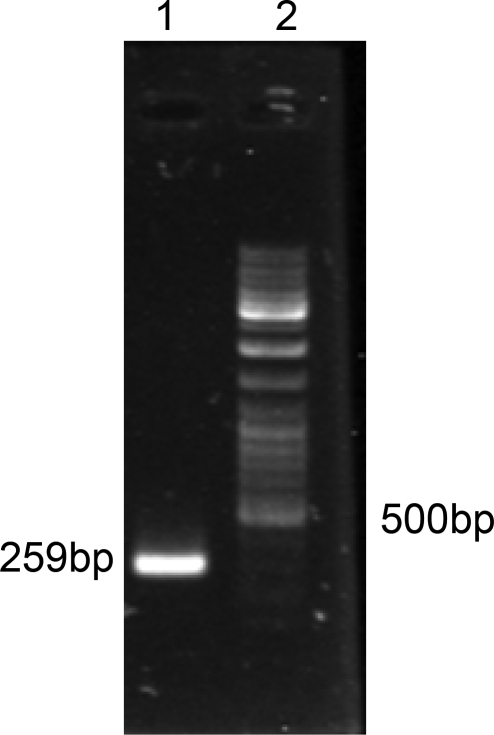
Lane1: 259bp of G1 strain of *Ecinococcus granulosus*, Lane2: DNA marker

Figure 1 shows a band of 259 bp representing the PCR product of extracted DNA from protoscoleces, which approves the Iranian G1 strain.

**Fig. 2:**
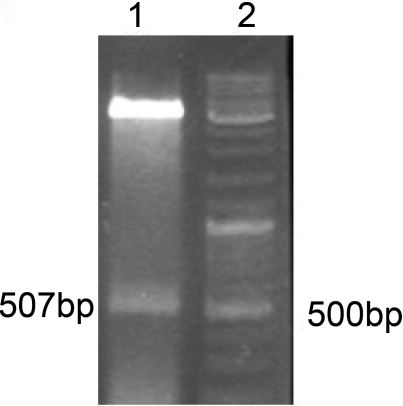
Lane1: 507bp fragment released from recombinant plasmid, Lane2: DNA marker

After subcloning of genes B and HydI into PET-Duet expression vector the recombinant plasmid was confirmed by restriction analysis (Fig. 2).

**Fig. 3:**
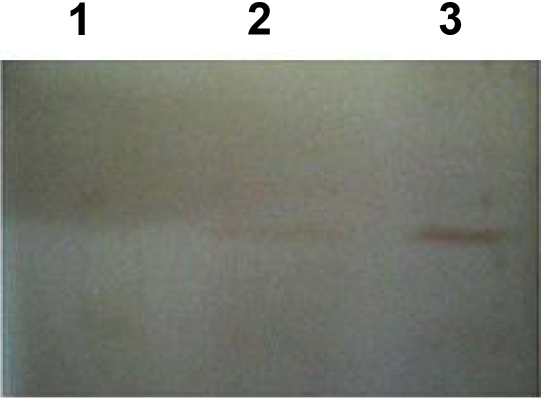
Western blotting of recombinant protein Lane1: Bacterial lysate without plasmid, lane2: bacterial lysate containing plasmid without recombinant protein, lane3: bacterial lysate containing recombinant protein

Recombinant expression vector was induced by IPTG and bacterial samples were taken intervals. Bacterial samples were lysed and analyzed by SDS - PAGE. Figure 3 shows the analysis of protein by Western Blot. Recombinant protein was purified by affinity chromatography using His-Tag column. After purification, recombinant protein was confirmed by western blot analysis using His Tag monoclonal antibody or hydatid positive human serum.

### ELISA test

**Fig. 4:**
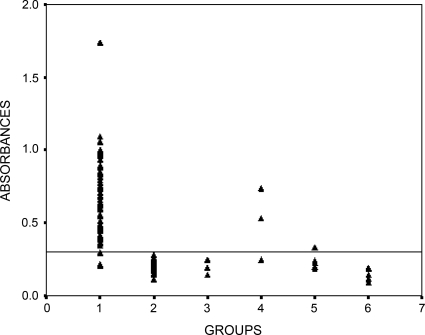
IgG-ELISA analysis of sera from patients infected with hydatidosis. Serum samples obtained from patients with hydatidosis (n=72, lane 1), control human sera (n=48, lane 2), toxoplasmosis (n=4, lane 3), strongyloidosis (n=4, lane 4), kala-azar (n=5, lane 5) and tuberculosis (n=5, lane 6)

Figure 4 demonstrates the analysis of all collected sera as stated in the Materials and Methods section. Accordingly, three cases of false positive including 2 cases pertaining to *S. stercoralis* infection, and one to kalaazar were detected with regards to analyzing the rAgB16. In addition, five cases of false negative were demonstrated. Based on these findings the sensitivity, specificity; positive and negative predictive values were calculated as 93.5%, 95.6%, 96% and 92.9%, in that order. The cut-off point was detected as 0.3 regarding rAgB16.

## Discussion

The issue of accurate diagnosis of hydatidosis is still obvious. The problem is mainly because there is no established gold standard to diagnose the disease, although in many parts of the world ELISA test using native AgB or even hydatid cyst fluid is widely used. Moreover, many immunological tests such as IFA, IHA, western blot, CFT, dot-ELISA etc have been utilized so far (18–23). In many cases, the physician prefers to confirm the disease via these tests and primarily uses of imaging techniques to reach an elementary diagnosis. Immunological tests more or less assist the physician to reach a conclusive diagnosis so evaluation and establishment an accurate and authentic diagnostic test is of high priority. One of the most convenient antigens has been used to implement ELISA test was recombinant antigens (24–28).

Following the challenge of producing and evaluating different recombinant antigens form *E. granulosus*, Kalantri et al. exploited the accession no. **DQ835667**, and produced recombinant EgAgB (24 kDa) which was more related to AgB4 (10). The antigen was tested by ELISA method and a sensitivity of 91.66% and specificity of 97.22% was reported accordingly. In another trial, conducted by our team, we produced recombinant antigen B1 subunit as EgAgB12 kDa, from *E. granulosus* (Iranian G1 isolates) in *E. coli* and evaluated it by ELISA (29). We could detect the sensitivity, specificity; positive and negative predictive values for this antigen as 96%, 97%, 97.2% and 95.5%, respectively. Of course, in comparison with native AgB we determined these parameters as 98.6%, 100%, 100%, and 98.5% for the latter antigen (29).

In a comprehensive study to evaluate different recombinant antigens, Virginio et al. used six recombinant antigens from *E. granulosus* including cytosolic maleate dehydrogenase isoform (EgcMDH), two 8 kDa subunits of antigen B (AgB8/1 and AgB8/2), an EF-hand calcium binding protein (EgCaBP2), a full-length (EgAFFPf) and a truncated form (EgAFFPt, aa 261-370) of an actin filament fragmenting protein. AgB8/2 was significantly higher than other antigens in sensitivity and specificity (30). Rott et al. found that rEgAgB8/2 was more effective than rEgAgB8/1 or native AgB in serodiagnosis of human hydatidosis (12). Besides the utilizing of recombinant antigens, subunits of AgB including antigens of 8, 16 and 24 kDa have been challenged by immunological tests. De La Rue et al. reported the sensitivity of 60.0% and 57.1% for 8- and 16-kDa subunits, respectively and 85.7% for 24-kDa subunit via immunoblot test (31). Sarkari et al. accounted the sensitivity of 80% for 8kDa and 72.5% for both 16 and 24 kDa and specificity of 100% for all three antigens using immunoblot test (23). A sensitivity of 80% and specificity of 100% for 8/12,16 kDa native subunits of AgB was reported by Hanilo et al. using immunoblot test (32). Comparing the findings of the studies conducted by our team witnesses altogether the priority of native AgB pertaining to diagnostic parameters by ELISA followed by rAgB8, cystic hydatid fluid, and rAgB16 (Table 1).

**Table 1:** Comparison of diagnostic parameters conducted by ELISA as for different evaluated antigens

Antigen	Sensitivity (%)	Specificity (%)	Positive PV (%)	Negative PV (%)	Reference
**rAgB 12 kDa**	96	97	97.2	95.5	(29)
**rAgB 16 kDa**	93.5	95.6	96	92.9	Present paper
**Native AgB**	98.6	100	100	98.5	(29)
**Cystic hydatid fluid**	96	97	97.2	95.5	(29)

The limitation of the study is that the number of utilized sera to detect the specificity of the test is not acceptable in terms of statistic analysis. We hope that in future by collecting more sera, can improve this defect. Considering that there are very limited data on preparing recombinant antigen of 16-kDa subunit of AgB, the present study might be able to elucidate the dark angels of the gap of diagnosing hydatidosis by serological tests.

In conclusion, considering all reported findings on evaluating the diagnostic tests of hydatidosis shows that still we need more investigations to reach a conclusive deduction. Native and recombinant antigens have their specific weak and strong points. It is up to the researchers in this arena to find and establish a gold standard method to diagnose hydatidosis as precisely as possible.
